# Neck Circumference Predicts Mortality in Hospitalized COVID-19 Patients

**DOI:** 10.3390/idr13040096

**Published:** 2021-12-10

**Authors:** Stefano Di Bella, Verena Zerbato, Gianfranco Sanson, Erik Roman-Pognuz, Paolo De Cristofaro, Andrea Palermo, Michael Valentini, Ylenia Gobbo, Anna Wladyslawa Jaracz, Elizabeta Bozic Hrzica, Cristiane Campello Bresani-Salvi, Alexandre Bezerra Galindo, Sergio Crovella, Roberto Luzzati

**Affiliations:** 1 Clinical Department of Medical, Surgical and Health Sciences, Trieste University, 34149 Trieste, Italy; gsanson@units.it (G.S.); romanpognuz.erik@gmail.com (E.R.-P.); roberto.luzzati@asugi.sanita.fvg.it (R.L.); 2 Infectious Diseases Unit, Trieste University Hospital (ASUGI), 34125 Trieste, Italy; verena.zerbato@gmail.com (V.Z.); michael.valentini@asugi.sanita.fvg.it (M.V.); ylenia.gobbo@gmail.com (Y.G.); annawladyslawa.jaracz@asugi.sanita.fvg.it (A.W.J.); elizabeta.bozichrzica@asugi.sanita.fvg.it (E.B.H.); 3 Clinical Endocrinology and Metabolism Outpatient Clinic, 64021 Giulianova, Italy; pdecristofaro@gmail.com; 4 Unit of Endocrinology and Diabetes, University Campus Bio-Medico, 00128 Rome, Italy; a.palermo@unicampus.it; 5 Laboratory of Virology and Experimental Therapy, Oswaldo Cruz Foundation, Ministry of Health, Recife 50070-902, Brazil; cristiane.bresani@cpqam.fiocruz.br; 6 School of Health and Life Science, Catholic University of Pernambuco, Recife 50070-902, Brazil; alexandre.galindo@unicap.br; 7 Department of Biological and Environmental Sciences, College of Arts and Sciences, Qatar University, Doha 2713, Qatar; crovelser@gmail.com

**Keywords:** neck, neck circumference, COVID-19, mortality, BMI, metabolic syndrome

## Abstract

We aimed to determine whether neck circumference predicts mortality among hospitalized COVID-19 patients with respiratory failure. We performed a prospective multicenter (Italy and Brasil) study carried out from March to December 2020 on 440 hospitalized COVID-19 patients with respiratory failure. Baseline neck circumference was measured. The study outcome was 30- and 60-days mortality. Female and male participants were classified as “large neck” when exceeding fourth-quartile. Patients had a median age of 65 years (IQR 54–76), 68% were male. One-quarter of patients presented with grade-1 or higher obesity. The median neck circumference was 40 cm (IQR 38–43): 38 cm (IQR 36–40) for female and 41 cm (IQR 39–44) for male subjects. “Large neck” patients had a significantly higher prevalence of hypertension (63 vs. 48%), diabetes (33 vs. 19%), obesity (26 vs. 14%), and elevated C-reactive protein (CRP) (98 vs. 88%). The cumulative mortality rate was 13.1% (*n* = 52) and 15.9% (*n* = 63) at 30 and 60 days, respectively. After adjusting for age, BMI, relevant comorbidities, and high C-reactive protein to albumin ratio, “large neck” patients showed a significantly increased risk of death at 30- (adjusted HR 2.50; 95% CI 1.18–5.29; *p* = 0.017) and 60-days (adjusted HR 2.26; 95% CI 1.14–4.46; *p* = 0.019). Neck circumference is easy to collect and provides additional prognostic information to BMI. Among hospitalized COVID-19 patients with respiratory failure, those with large neck phenotype had a more than double risk of death at 30 and 60 days.

## 1. Introduction

The SARS-CoV-2 pandemic, beginning in Wuhan (China) in December 2019, is still creating huge difficulties for the healthcare services of several countries worldwide. The clinical manifestations of the coronavirus disease 2019 (COVID-19) are heterogeneous, ranging from mild diseases to extensive pulmonary disease manifesting as acute respiratory distress syndrome which may lead to death. Despite the availability of new therapeutic options, COVID-19 mortality remains high [1], especially in hospitalized patients with pneumonia who require oxygen therapy support. Severe and fatal COVID-19 episodes are more prevalent among elderly individuals with cardiometabolic and respiratory comorbidities such as hypertension, chronic lung disease and diabetes [2,3]. Notably, most of these concomitant diseases constitute the definition of metabolic syndrome, a common metabolic disorder in the general population. In terms of COVID-19 treatment, at the present time, the most effective drug in terms of mortality reduction is dexamethasone, able to reduce the inflammatory immune-mediated pathogenic process that characterizes the second phase of the disease [4]. Heparin use at therapeutic doses did not show encouraging results, possibly for a timing issue or for the intrinsic characteristics of COVID-19 pulmonary thrombi (phlogistic platelet-rich “white thrombi’’) [5]. It has been demonstrated that hormonal and metabolic features have a significant impact on the clinical progression of COVID-19 patients. In fact, male subjects with COVID-19 have a higher mortality risk than female subjects, with 60–70% of COVID-19 associated deaths occurring in men [6]. In addition, from a metabolic point of view, it is well known that the body mass index (BMI) correlates with a worse prognosis among COVID-19 patients [7], however, BMI measurement as a prognostic tool has some limitations since it disregards the fat distribution. In fact, more than the mere BMI, is the fat distribution to reflect a dysmetabolism oriented toward metabolic syndrome. Current findings confirm that upper body fat is a good predictor of metabolic syndrome [8] with several studies demonstrating upper-body obesity to have a stronger association with cardiometabolic conditions compared to lower-body obesity. Patients with a central obesity distribution have a higher risk of hypertension, type 2 diabetes, obstructive sleep apnea syndrome, and non-alcoholic fatty liver diseases. Metabolic syndrome is a pathologic condition characterized by abdominal obesity, insulin resistance, hypertension, and hyperlipidemia. The precise global prevalence of metabolic syndrome is difficult to measure but it is estimated to be about one-quarter of the world population [9]. It is well known that metabolic syndrome is associated with an increase in pro-inflammatory cytokines (IL-6, TNF-α), markers of pro-oxidant status (OxLDL, uric acid), and prothrombotic factors (PAI-1) [10]. Moreover, C-reactive protein (CRP) strongly correlates with upper body fat [8] and neck circumference is associated with upper body fat (central body obesity) [11]. In addition, neck circumference has been shown to be strongly related to insulin resistance, early-stage atherosclerosis, diabetes, coronary heart diseases, and cardiometabolic syndrome even after adjustment for visceral adipose tissue and BMI [12]. In a previous study on 132 COVID-19 patients, we demonstrated that neck circumference measured on admission is independently and significantly associated with the progression to mechanical ventilation (adjusted OR 1.26-per 1 cm increase; 95% CI: 1.120–1.417; *p* < 0.001) [13]. At that time our sample was too small to investigate the relationship between neck circumference and mortality. In the present study, with a larger sample size, we aimed to assess if neck circumference measured on hospital admission correlates with the occurrence of short- and mid-term mortality among COVID-19 hospitalized patients, thus investigating its potential as a cheap and easy-to-use prognostic predictor.

## 2. Materials and Methods

To further expand our previous results, we performed a multicenter prospective study on patients admitted to the Infectious Diseases and Intensive Care Unit of Trieste University hospital (Italy) and the Intensive Care Unit of Recife Hospital (Brazil) for COVID-19 pneumonia with respiratory failure from March to December 2020. Nasopharingeal swab samples were obtained from all patients and tested using real-time reverse transcriptase-polymerase chain reaction assays. COVID-19 pneumonia was diagnosed in the whole study population according to the World Health Organization guidance. Subjects with advanced cancer, or prognosis <3 months for diseases other than COVID-19 were excluded from the analysis. Following hospital admission, all patients required oxygen therapy. During hospital stay, patients underwent non-invasive and/or invasive mechanical ventilation according to their clinical conditions. Patients were followed for 60 days. On admission, demographics, clinical and anthropometric data were collected. Demographic collected data were: age and gender. Comorbidity collected data were: Body (1) mass index ≥ 35 kg/m^2^; (2) arterial hypertension; (3) heart disease and (4) diabetes mellitus. Baseline neck circumference was measured with an inextensible measuring tape immediately below the laryngeal prominence and perpendicular to the long axis of the neck [9]. In addition, other clinical and laboratoristic parameters were recorded, namely: BMI, lymphocyte count, C-reactive protein (CRP), serum albumin, and d-dimer levels. The association between neck circumference and mortality, defined as death from any cause within 30 and 60 days, was investigated. The study was conducted in compliance with the Declaration of Helsinki and the International Conference on Harmonization Principles of Good Clinical Practice and approved by the Ethics Committee of the participating Hospitals.

Based on an expected prevalence of “large neck” phenotype patients ranging from 30% to 54% according to previously published studies [10,11,12], and assuming a confidence level of 95% (z^2^ = 1.96), and a 5% margin of error (e), a minimum required sample size of 323–382 patients was calculated a priori based on the following equation: n = [z^2^ × p × (1−p)]/e^2^. Participants were categorized based on neck circumference quartiles and a “large neck” phenotype was identified based on the sex-specific upper quartile (Q4). Unadjusted comparisons between patients belonging to neck circumference Q4 or Q1–Q3 with the other categorical study variables were performed. The difference in death time between Q4 versus Q1–Q3 NC groups was explored. Observations were right-censored until 30 or 60 days from hospital admission (known survival), respectively. Both unadjusted and adjusted analyses were performed. Crude evaluation was carried out by comparing Kaplan–Meier curves and survival differences between neck circumference groups were assessed with the log-rank Mantel–Cox test. Multivariate Cox regression models were run to estimate patient’s risk of death at different time intervals according to the neck circumference groups, adjusted for presence of obesity (BMI ≥ 30 kg/m^2^) and variables significantly related to mortality (*p*-value < 0.05) in the bivariate analyses. The stepwise method was adopted (*p*-values: entry 0.05; removal 0.10) to identify the best subset of predictors for each considered mortality interval. The results were presented as adjusted proportional hazard ratios with 95% confidence intervals and with either crude or adjusted Kaplan–Meier curves, as appropriate. The assumption of each multivariable Cox model was assessed by comparing the log–log transformation versus the log of survival time of the Kaplan–Meier curves for the neck circumference groups. The curves presented an approximately parallel trend without intersecting, consequently, a constant over-time proportional hazard was assumed. Statistical analyses were performed using the software IBM SPSS Statistics, version 24.0. For all tests, an alpha level of *p* < 0.05 was set for statistical significance.

## 3. Results

During the study period, 440 patients were admitted to the study wards. After excluding 43 subjects with severe comorbid conditions, 397 patients constituted the study population (median age 65 years, IQR 54–76; male 272, 68.5%). The main characteristics of the enrolled patients are described in Table 1. One patient out of four (*n* = 99; 25%) presented grade-1 or higher obesity. The median neck circumference of the whole study population was 40 cm (IQR 38–43), 38 cm (IQR 36–40) for female, and 41 cm (IQR 39–44) for male subjects. Neck circumference showed a moderate positive correlation with BMI (Pearson’s *r* = 0.617; *p* < 0.001). Accordingly, female and male participants were classified as “large neck” when exceeding the neck circumference Q4 threshold, i.e., 40 cm or 44 cm, respectively. In “large neck” patients, a statistically significant higher prevalence of arterial hypertension (63 vs. 48%), diabetes (33 vs. 19%), obesity (26 vs. 14%), and high CRP levels (98 vs. 88%) was found (Table 1).

**Figure 1:**
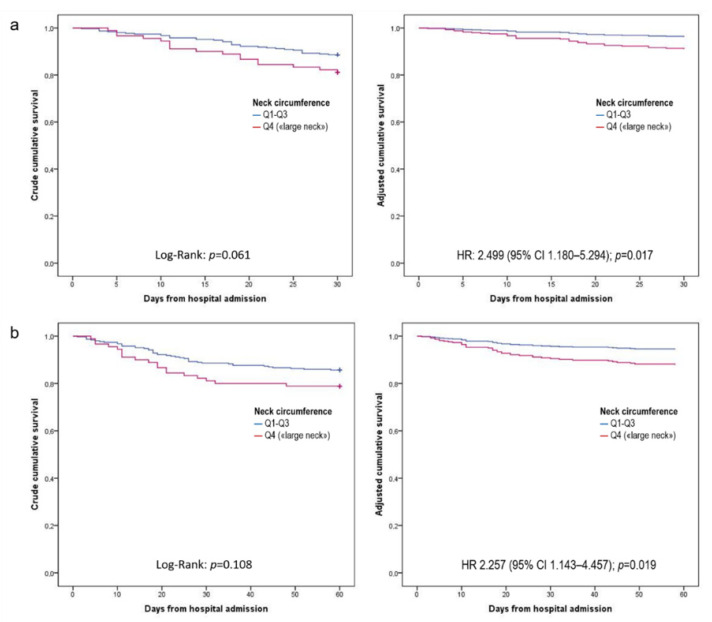
Crude and adjusted Kaplan–Meier curves for 30- (**a**) and 60-days (**b**) mortality according to the neck circumference.

In the bivariate analysis, both 30- and 60-days mortality were significantly associated with older age, hypertension, heart disease, diabetes, low albumin levels and high CRP to albumin ratio (CAR) (Table 2). Mortality data within 2 months from hospital admission were available for the whole population. The cumulative mortality rate was 13.1% (*n* = 52) and 15.9% (*n* = 63) at 30 and 60 days, respectively. Results of unadjusted and adjusted survival analyses are reported in Table 3; crude and adjusted 30- and 60-days survival curves are reported in Figure 1. In unadjusted analyses, patients with a “large neck” phenotype showed only a non-statistically significant trend (*p* = 0.061) towards a higher 30-days risk of death in crude survival analysis compared to those without this condition, while the difference was not statistically significant for 60-days mortality. After adjusting for age, BMI, relevant comorbidities, and high CAR, “large neck” phenotype patients showed a statistically significant increased risk of death at 30- (adjusted HR 2.50; 95% CI 1.18–5.29; *p* = 0.017) and 60-days (adjusted HR 2.26; 95% CI 1.14–4.46; *p* = 0.019). Figure 1 provides a graphical illustration of crude and adjusted Kaplan–Meier curves for 30- and 60-days mortality according to the neck circumference. Similar results were obtained by testing different proportional hazard models, after including sex instead of BMI (adjusted HR 1.99; 95% CI 1.08–3.66: *p* = 0.028) or in addition to BMI (adjusted HR 2.26; 95% CI 1.14–4.46; *p* = 0.019) among confounders.

## 4. Discussion

Our results support the evidence that dysmetabolism is a factor that contributes to COVID-19 pathogenesis. Indeed, the pathogenesis of this new viral disease seems to be more related to a dysregulated immune and inflammatory response than to direct viral tissue damage. The association between dysmetabolism (e.g., metabolic syndrome) and baseline inflammation and prothrombotic state is supported by robust data. In terms of “prothrombotic state”, it has also been demonstrated that venous and pulmonary thromboembolism are common complications of COVID-19 (approximately 30% of hospitalized patients) with a significant impact on clinical outcome. Many studies demonstrated that the specific patterns of “excess fat distribution” conferred different metabolic risks, pointing out the pro-inflammatory role of visceral adipose tissue and upper body adiposity. When facing infectious disorders, the fat excess can impair the immune system response through a chronic basal inflammatory status and it has been hypothesized that specific fat depots could increase the vascular damage [14,15]. Males are more susceptible to upper-body fat accumulation, due to sex hormone differences and males are more susceptible also to a complicated/fatal COVID-19 clinical progression. Neck circumference is an emerging anthropometric parameter that has been proposed to reflect human metabolic health [16]. Neck circumference is considered a proxy for central body obesity, which is considered the “most harmful” obesity. In our cohort, in the present study, patients with a “large neck” phenotype had a statistically significant higher prevalence of arterial hypertension, diabetes, obesity, and high CRP levels. In the present investigation, only a moderate—although statistically significant—correlation between BMI and NC was found, meaning that BMI cannot substitute NC, and vice-versa. However, compared to BMI, NC may be a more accurate and easy-to-measure marker of adiposity and metabolic risk in daily clinical practice (e.g., weighing a patient under mechanical ventilation may not be simply feasible, measuring her/his height while lying in a bed may lead to more imprecise data than gauging an NC). Moreover, we believe that neck circumference could outperform the prognostic predictive value of BMI among COVID-19 patients, being more associated with a specific fat distribution pattern, thus reflecting a “bad” obesity distribution pattern. Interestingly, a large neck circumference has been significantly associated with the prevalence of obstructive sleep apnea syndrome [17]. According to the literature data, obstructive sleep apnea syndrome occurs in 2–4% of adults, increasing by 2.5 times the risk of sudden death. When looking at SARS-CoV-2-infected patients, a recent study showed that there was an increased risk of mortality in COVID-19 patients with obstructive sleep apnea syndrome (OR = 2.59; 95% CI 1.218–5.507) independently from BMI, male gender, age, diabetes, cardiovascular diseases, and obstructive lung disease [17]. It is possible that part of the risk is attributable to the fact that, since gasping is a common phenomenon in patients with obstructive sleep apnea syndrome, this can predispose to aspiration of viral particles [18]. Furthermore, also other mechanical problems in obstructive sleep apnea syndrome contribute to less effective ventilation treatment, including a higher risk of difficult intubation and non-invasive ventilation may be less effective due to the intermittent airway obstruction episodes [19]. In addition, higher levels of inflammatory markers have been reported in obstructive sleep apnea syndrome patients compared to patients without obstructive sleep apnea syndrome [20]. This could increase the risk for overproduction of early response proinflammatory cytokines which could increase the chances of developing cytokine storm syndrome [21]. In our series, COVID-19-hospitalized patients with respiratory failure and a large neck phenotype had a more than double risk of death (both at 30 and 60 days). Given the doubts on the best predictive measure, including both NC and BMI in the multivariable model despite a potential collinearity problem, showed that NC seemed to outperform BMI in predicting mortality. Although we were confident to have reduced this risk by inserting BMI and NC as categorical variables, this aspect should be considered when interpreting the results of the first regression analysis. However, NC confirmed its predictive power in further models run after excluding BMI.

Our results support the rationale that a large neck is associated with a premorbid increased proinflammatory and prothrombotic status, which makes the patient more prone to progress toward an unfavorable outcome. Neck circumference is an independent predictor for mortality in our hospitalized COVID-19 patients with respiratory failure and should complement the baseline evaluation of such patients.

## Figures and Tables

**Table 1:** Main demographics and clinical characteristics of patients presenting or not a “large neck” (sex-specific neck circumference exceeding the 3rd quartile of the study population).

	Full Population	“Large Neck”		*p*-Value
		No	Yes	
Demographics				
Age (years)				0.619
18–54	113 (28.5%)	82 (26.7%)	21 (23.3%)	
55–65	101 (25.4%)	74 (24.1%)	26 (28.9%)	
66–76	97 (24.4%)	74 (24.1%)	25 (27.8%)	
>76	86 (21.7%)	77 (25.1%)	18 (20.0%)	
Sex (male)	272 (68.5%)	210 (68.4%)	62 (68.9%)	0.931
Comorbid conditions				
Body mass index ≥ 35 kg/m^2^ [375]	32 (8.1%)	7 (2.4%)	25 (29.1%)	<0.001
Arterial hypertension	205 (51.6%)	148 (48.4%)	57 (63.3%)	0.012
Heart disease	105 (26.4%)	80 (26.1%)	25 (27.8%)	0.758
Diabetes mellitus	90 (22.7%)	60 (19.5%)	30 (33.3%)	0.006
Biochemical tests				
Lymphocytes < 1500/μL [392]	345 (88.0%)	263 (86.8%)	82 (92.1%)	0.173
D-dimer > 500 ng/mL FEU [371]	298 (80.3%)	226 (79.3%)	72 (83.7%)	0.366
Albumin < 3.5 g/dL [378]	237 (62.7%)	187 (63.6%)	50 (59.5%)	0.495
C-reactive protein ≥ 1.0 mg/dL [387]	349 (90.2%)	262 (87.9%)	87 (97.8%)	0.003
CRP-to-albumin ratio > 56.6 [369]	54 (14.6%)	38 (13.3%)	16 (19.3%)	0.174

Data are reported as number (percentage). Numbers in squared brackets reports the total number of available data when missing data are present. FEU: fibrinogen equivalent units. CRP: C-reactive protein.

**Table 2:** Association of study variables with 30- and 60-days mortality.

	30-Days Follow-Up			60-Days Follow-Up		
	Survived	Dead	*p*-Value	Survived	Dead	*p*-Value
**Demographics**						
Age > 76 years	66 (19.1%)	29 (55.8%)	<0.001	60 (18.0%)	35 (55.6%)	<0.001
Sex (male)	240 (69.6%)	32 (61.5%)	0.245	233 (69.8%)	39 (61.9%)	0.218
**Comorbid conditions**						
Arterial hypertension	170 (49.3%)	35 (68.6%)	0.010	160 (47.9%)	45 (72.6%)	<0.001
Heart disease	74 (21.4%)	31 (60.8%)	<0.001	69 (20.7%)	36 (58.1%)	<0.001
Diabetes mellitus	67 (19.4%)	23 (44.2%)	<0.001	64 (19.2%)	26 (41.3%)	<0.001
**Biochemical tests**						
Lymphocytes < 1500/μL	303 (88.9%)	42 (82.4%)	0.182	293 (88.8%)	52 (83.9%)	0.274
D-dimer > 500 ng/mL FEU	265 (79.1%)	33 (91.7%)	0.079	259 (79.2%)	39 (88.6%)	0.097
Albumin < 3.5 g/dL	197 (59.7%)	40 (83.3%)	0.002	189 (59.2%)	48 (81.4%)	0.001
C-reactive protein ≥ 10 mg/L	309 (90.4%)	40 (88.9%)	0.789	301 (90.7%)	48 (87.3%)	0.434
CRP-to-Albumin ratio > 56.6	40 (12.2%)	14 (34.1%)	<0.001	39 (12.3%)	15 (29.4%)	0.001

Data are reported as number (percentage). FEU: fibrinogen equivalent units. CRP: C-reactive protein.

**Table 3:** Variables predictive for mortality in bivariate and multivariate survival analyses.

Dependent Variable	Predictor	Unadjusted Risk ^a^ χ^2^; *p*-Value	Adjusted Risk ^b^ HR (95% CI); *p*-Value
**30-day mortality**	“Large neck” phenotype	3.515; *0.061*	2.499 (1.180–5.294); *0.017*
	Age > 77 years	/	7.570 (3.309–17.317); *<0.001*
	CRP-to-Albumin ratio > 56.6	/	2.620 (1.248–5.500); *0.011*
	Heart disease	/	2.601 (1.246–5.428); *0.011*
**60-day mortality**	“Large neck” phenotype	2.585; *0.108*	2.257 (1.143–4.457); *0.019*
	Age > 77 years	/	6.547 (3.209–13.356); *<0.001*
	CRP-to-Albumin ratio > 56.6	/	2.220 (1.115–4.419); *0.023*
	Heart disease	/	2.338 (1.213–4.510); *0.011*

^a^: Log-rank Mantel–Cox test. ^b^: multivariable forward stepwise Cox regression analysis; predictors included in the regression models: neck circumference, age, CRP-to-albumin ratio, BMI, hypertension, diabetes, heart disease; predictors not reported in the table were excluded by the forward stepwise method from the respective final models. CI: confidence interval. CRP: C-reactive protein. BMI: body mass index.

## Data Availability

Electronics protected datasets.

## References

[1] Thomas K.S., Zhang W., Dosa D.M., Carder P., Sloane P., Zimmerman S. (2021). Estimation of Excess Mortality Rates Among US Assisted Living Residents During the COVID-19 Pandemic. JAMA Netw. Open.

[2] Guo W., Li M., Dong Y. (2020). Diabetes is a risk factor for the progression and prognosis of COVID-19. Diabetes Metab. Res. Rev..

[3] Carter S.J., Baranauskas M.N., Fly A.D. (2020). Considerations for obesity, vitamin D, and physical activity amid the COVID-19 pandemic. Obesity.

[4] Sterne J.A.C., Murthy S., Diaz J.V., Slutsky A.S., Villar J., Angus D.C., Annane D., Azevedo L.C.P., Berwanger O., Cavalcanti A.B. (2020). WHO Rapid Evidence Appraisal for COVID-19 Therapies (REACT) Working Group. Association Between Administration of Systemic Corticosteroids and Mortality Among Critically Ill Patients With COVID-19: A Meta-analysis. JAMA.

[5] REMAP-CAP Investigators, ACTIV-4a Investigators, ATTACC Investigators (2021). Therapeutic Anticoagulation with Heparin in Critically Ill Patients with COVID-19. N. Engl. J. Med..

[6] Antonello R.M., Dal Bo E., De Cristofaro P., Luzzati R., Di Bella S. (2020). The seXY side of COVID-19: What is behind female protection?. Infez. Med..

[7] Chowdhury A.I., Alam M.R., Rabbi M.F., Rahman T., Reza S. (2021). Does higher body mass index increase COVID-19 severity? A systematic review and meta-analysis. Obes. Med..

[8] Grundy S.M., Williams C., Vega G.L. (2018). Upper body fat predicts metabolic syndrome similarly in men and women. Eur. J. Clin. Investig..

[9] Saklayen M.G. (2018). The global epidemic of the metabolic syndrome. Curr. Hypertens. Rep..

[10] Srikanthan K., Feyh A., Visweshwar H., Shapiro J.I., Sodhi K. (2016). Systematic Review of Metabolic Syndrome Biomarkers: A Panel for Early Detection, Management, and Risk Stratification in the West Virginian Population. Int. J. Med. Sci..

[11] Tal S., Litovchik I., Klar M.M., Maresky H.S., Grysman N., Wiser I., Vitkon-Barkay I., Marcus G., Tzuman O., Pereg D. (2019). The association between neck adiposity and long-term outcome. PLoS ONE.

[12] Preis S.R., Massaro J.M., Hoffmann U., D’Agostino Sr R.B., Levy D., Robins S.J., Meigs J.B., Vasan R.S., O’Donnell C.J., Fox C.S. (2010). Neck circumference as a novel measure of cardiometabolic risk: The framingham heart study. J. Clin. Endocrinol. Metab..

[13] Di Bella S., Cesareo R., De Cristofaro P., Palermo A., Sanson G., Roman-Pognuz E., Zerbato V., Manfrini S., Giacomazzi D., Dal Bo E. (2021). Neck circumference as reliable predictor of mechanical ventilation support in adult inpatients with COVID-19: A multicentric prospective evaluation. Diabetes Metab. Res. Rev..

[14] Lim S., Meigs J.B. (2013). Ectopic fat and cardiometabolic and vascular risk. Int. J. Cardiol..

[15] Lim S., Meigs J.B. (2014). Links between ectopic fat and vascular disease in humans. Arterioscler. Thromb. Vasc. Biol..

[16] Assyov Y., Gateva A., Tsakova A., Kamenov Z. (2017). A comparison of the clinical usefulness of neck circumference and waist circumference in individuals with severe obesity. Endocr. Res..

[17] Voncken S.F.J., Feron T.M.H., Laven S.A.J.S., Karaca U., Beerhorst K., Klarenbeek P., Straetmans J.M.J.A.A., de Vries G.J., Kolfoort-Otte A.A.B., de Kruif M.D. (2021). Impact of obstructive sleep apnea on clinical outcomes in patients hospitalized with COVID-19. Sleep Breath..

[18] Beal M., Chesson A., Garcia T., Caldito G., Stucker F., Nathan C.O. (2004). A pilot study of quantitative aspiration in patients with symptoms of obstructive sleep apnea: Comparison to a historic control group. Laryngoscope.

[19] Nagappa M., Wong D.T., Cozowicz C., Ramachandran S.K., Memtsoudis S.G., Chung F. (2018). Is obstructive sleep apnea associated with difcult airway? Evidence from a systematic review and meta-analysis of prospective and retrospective cohort studies. PLoS ONE.

[20] Nadeem R., Molnar J., Madbouly E.M., Nida M., Aggarwal S., Sajid H., Naseem J., Loomba R. (2013). Serum infammatory markers in obstructive sleep apnea: A meta-analysis. J. Clin. Sleep Med..

[21] Jose R.J., Manuel A. (2020). COVID-19 cytokine storm: The interplay between infammation and coagulation. Lancet Respir. Med..

